# A Case Report on Extensive Arterial Thrombosis: A Rare Complication of COVID-19 Infection

**DOI:** 10.7759/cureus.15378

**Published:** 2021-06-01

**Authors:** Shobha Mandal, Sumit Gami, Sushmita Khadka, Barun Ray, Subash Ghimire

**Affiliations:** 1 Internal Medicine, Guthrie Robert Packer Hospital, Sayre, USA; 2 Medicine, Universal College of Medical Sciences, Bhairahawa, Bhairahawa, NPL; 3 Internal Medicine, Nidan Hospital, Lalitpur, NPL; 4 Internal Medicine, Bishweshwar Prasad Koirala Institute of Health Sciences, Dharan, NPL

**Keywords:** arterial clot, venous clot, coronavirus disease of 2019 (covid-19), coagulopathy, arterial thrombosis, left ventricle thrombus

## Abstract

Coronavirus disease (COVID-19) is a global health crisis leading to increased morbidity and mortality worldwide. It is associated with increased activation of the clotting system leading to thrombotic complications increasing the risk of life-threatening complications. We report a case of a 70-years-old COVID-19 positive patient who presented with both lower extremities and forearm pain. On workup, she was found to have an extensive arterial clot. In patients with COVID-19, arterial clots may be the initial presenting symptoms to the hospital and can be fatal if not brought to attention on time.

## Introduction

Coronavirus disease of 2019 (COVID-19) emerged as a dramatic health emergency leading to millions of deaths worldwide. It is mainly a respiratory tract disease presenting as shortness of breath, cough, fever, severe acute respiratory distress, and multiple organ failures. It also involved multiple other organs and systems of the body including vascular [1,2]. COVID-19 is associated with increased activation of the clotting system leading to thrombotic complications in 5% to 23% of cases [3]. It is known to cause significant venous and arterial clots leading to an increased risk of life-threatening complications like pulmonary embolism, myocardial infarction, ischemic stroke, splenic infarct, and left ventricular clot [4].

## Case presentation

**Figure 1:**
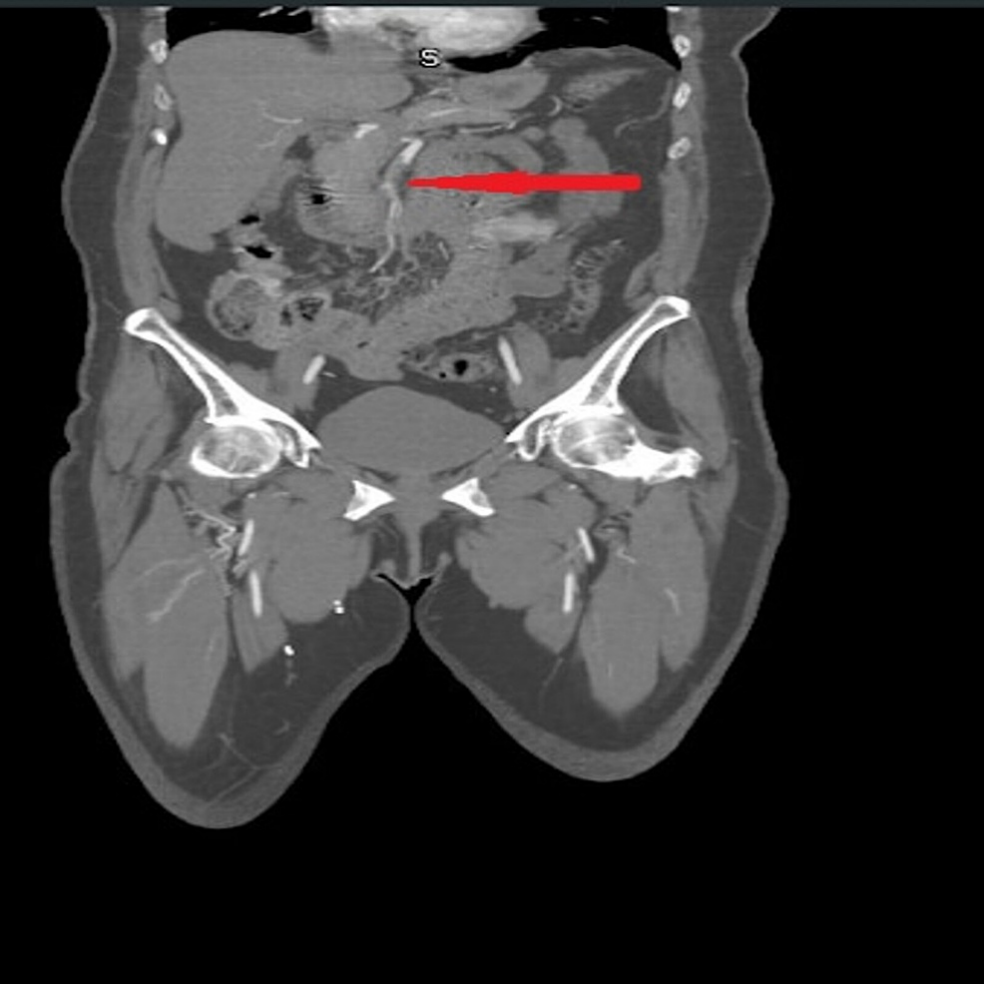
CT of abdomen and pelvis showing thrombosis of superior mesenteric artery

A 70-year-old female with a past medical history of coronary artery disease, status post-coronary artery bypass grafting, ventricular aneurysm resected during coronary artery bypass grafting, hypertension, Chronic Obstructive Pulmonary Disease Global Initiative for Chronic Obstructive Lung Disease stage 3 with cor-pulmonale, tested positive for COVID 19 three weeks earlier. She self-quarantined for three weeks but she started having worsening shortness of breath. She also noticed bilateral upper and lower extremities pain and swelling. She came to the emergency department for evaluation. Laboratory workup showed normal complete blood counts, comprehensive metabolic panel but inflammatory markers were elevated. Lactate dehydrogenase (LDH) was 1455 (135-214 U/L), and C-reactive protein was 5.4 (<0.5 mg/dL). Venous duplex of right upper extremity showed occlusion at the right distal brachial artery, and subsequent computed tomography (CT) angiography of abdomen, pelvis, and aorta demonstrated extensive arterial thrombosis of the superior mesenteric artery (Figure 1).

**Figure 2:**
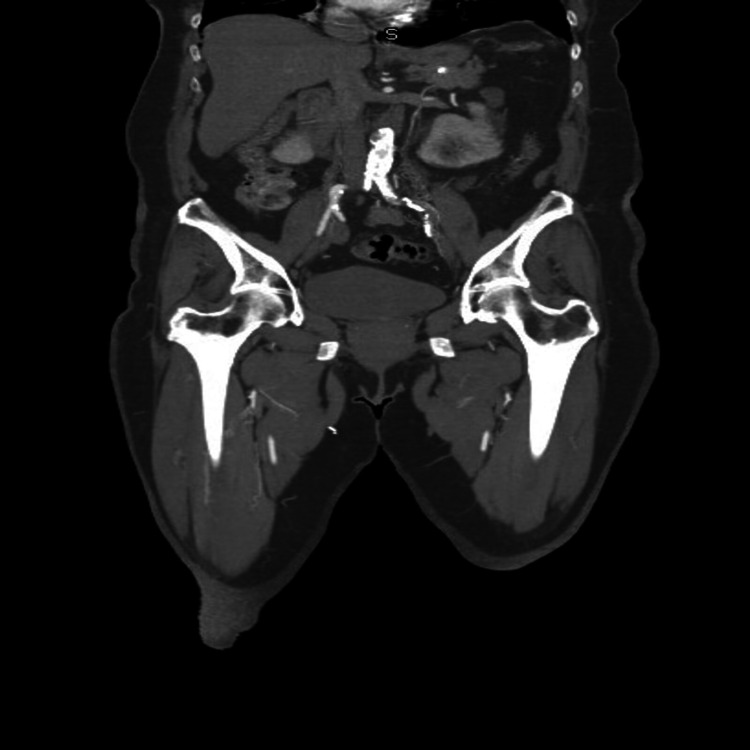
CT of abdomen and pelvis showing right and left iliac artery thrombosis

CT of the abdomen and pelvis demonstrated thrombosis in right and left common iliac artery (Figure 2).

**Figure 3:**
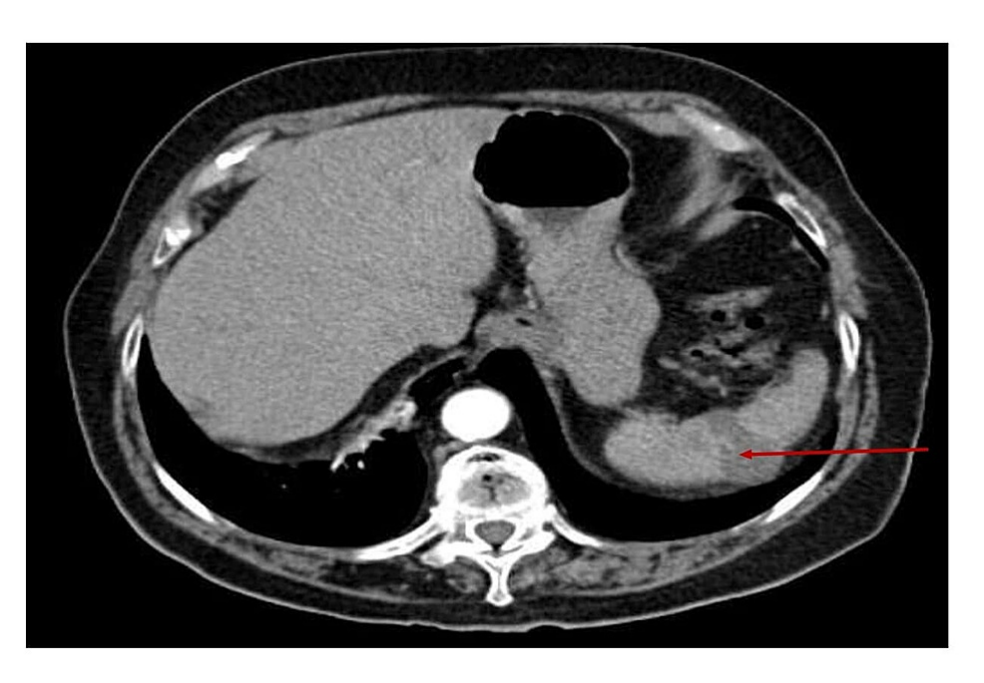
CT of abdomen and pelvis showing wedge-shaped infraction of spleen

It also showed an area of wedge-shaped infarction in the spleen (Figure 3).

**Figure 4:**
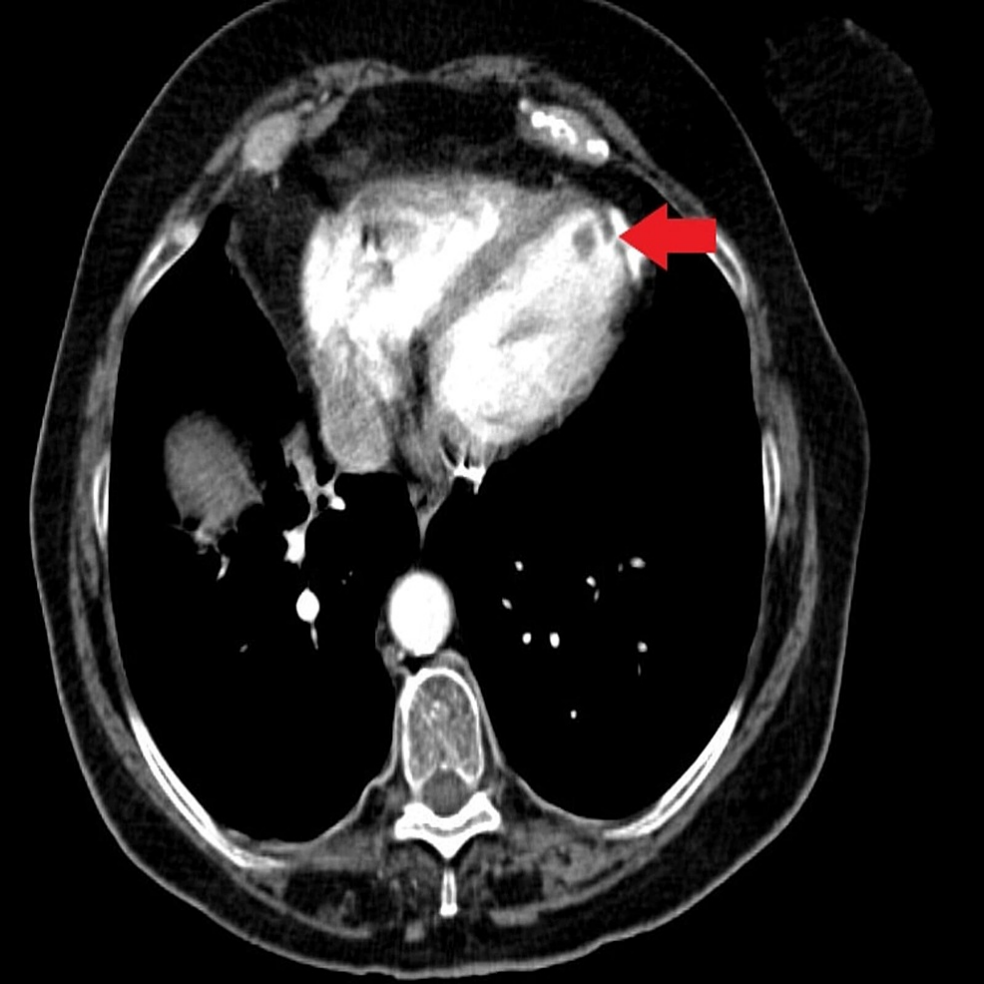
CT chest showing clot at the left ventricle

A thrombus at the apex of the left ventricle is shown in Figure 4.

Heparin drip was started initially, and later after multidisciplinary discussion with a hematologist, vascular surgeon, and cardiologist, her anticoagulation was changed to subcutaneous Enoxaparin Sodium 80 mg/0.8 ml twice daily. Further investigations including an arterial brachial index of both upper and lower extremities were normal. Because of severe left leg pain, magnetic resonance imaging (MRI) of the spine was obtained, which was negative for acute abnormality. She was managed medically and was discharged home. On her follow-up visit two weeks later, she reported improvement in her left leg pain and claudication. On the follow-up visit with hemato-oncologist two weeks later, further workup including JAK2, beta-2 glycoprotein antibodies, lupus anticoagulant anticardiolipin antibodies were done which came negative.

Since the extensive arterial clot was thought to be present in the setting of underlying COVID-19 infection, she was examined by a vascular surgeon and was advised to continue the anticoagulant for at least six months. The patient had a negative hypercoagulable workup, and the transient hypercoagulable state was more likely induced by COVID-19 infection, leading to micro and macrovascular thrombotic angiopathy.

## Discussion

The exact mechanisms that activate coagulation cascade in SARS-CoV-2 infection are still unknown, but they are associated with increased thrombogenesis. SARS-CoV-2 viral infection can activate the plasmatic clotting system by activating multiple procoagulant pathways. The viral infection itself can cause endothelial dysfunction leading to excess thrombin production resulting in a hypercoagulable state. Both extrinsic and intrinsic coagulation pathways including the platelets, mast cells, and factors XII are found to be activated in severe COVID-19 infections [5].

In COVID 19 infection, a massive systematic inflammations lead to cytokine storm leading to increased pro-inflammatory cytokines such as IL-6, TNF-α, CRP, ferritin, fibrinogen, which further leads to endothelial injury resulting in intimal necrosis and expression of adhesion molecules. The overexpressed adhesion molecule adheres with platelets leading to thrombocytopenia [6]. It can also lead to microcirculatory flow abnormalities leading to multiple clot formations within the capillaries, arterioles, and arteries like in our patient. The blockage of arteries can lead to circulatory dysfunction in a solid organ resulting in multiorgan failure and eventually death of the patient with COVID-19 infection [7].

Angiotensin-converting enzymes-2 are typically found on various cells such as lymphocytes, alveolar cells, monocytes/macrophages, and platelets. In SARS-CoV-2 infection, the surface S protein of SARS-CoV-2 binds to its target transmembrane receptor (ACE2 protein) and down-regulates the expression of ACE2 protein. It leads to angiotensin II accumulation in the body, which interacts with platelets and endothelial cells, resulting in further promotion of clot formation [8]. In addition to all these mechanisms, patients with severe hypoxia in COVID-19 pneumonia have enhanced clot formation because of the hypoxia-inducible transcription factor (HIF)-dependent signaling pathway leading to increased blood viscosity [9].

Our patient had a right distal brachial artery occlusion on a venous duplex scan. CT of the abdomen and pelvis showed extensive arterial thrombosis, including the distal abdominal aorta, left common iliac artery, superior mesenteric artery, and other sites leading to a deep arterial clot of multiple sites splenic infarct, and left ventricle thrombus formation. Patients suffering from COVID-19 have extensive systemic inflammation, hypoxia, pro and hyper coagulant states leading to extensive multiple site thrombosis like in our patient. Patients may sometimes present with extremity swelling and shortness of breath, which can be confused with congestive heart failure.

## Conclusions

COVID-19 is well known to cause respiratory failure and respiratory complications but in severe cases, it is found to cause thromboembolic events leading to arterial and/or venous clot formation. Patients with extensive clot burden are found to have poor outcomes. These patients need prompt treatment with anticoagulation and careful monitoring. Patients with extensive clot burden are at high risk of death hence any patient diagnosed with COVID-19, unless contraindicated must be kept on a prophylactic anticoagulant during the hospital stay to prevent them from forming a clot.
